# Epicardial adipose tissue and muscle distribution affect outcomes in very old patients after transcatheter aortic valve replacement

**DOI:** 10.1093/ehjopen/oeae073

**Published:** 2024-09-20

**Authors:** Susanne Rohrbach, Oezge Uluocak, Marieke Junge, Fabienne Knapp, Rainer Schulz, Andreas Böning, Holger M Nef, Gabriele A Krombach, Bernd Niemann

**Affiliations:** Institute of Physiology, Justus Liebig University Giessen, Aulweg 129, 35392 Giessen, Germany; Department of Cardiac and Vascular Surgery, University Hospital Giessen and Marburg, Justus Liebig University Giessen, Rudolph-Buchheim-Strasse 7, 35392 Giessen, Germany; Department of Cardiac and Vascular Surgery, University Hospital Giessen and Marburg, Justus Liebig University Giessen, Rudolph-Buchheim-Strasse 7, 35392 Giessen, Germany; Institute of Physiology, Justus Liebig University Giessen, Aulweg 129, 35392 Giessen, Germany; Institute of Physiology, Justus Liebig University Giessen, Aulweg 129, 35392 Giessen, Germany; Department of Cardiac and Vascular Surgery, University Hospital Giessen and Marburg, Justus Liebig University Giessen, Rudolph-Buchheim-Strasse 7, 35392 Giessen, Germany; Department of Cardiology, University Hospital Giessen and Marburg, Justus Liebig University Giessen, Klinikstrasse 33, 35392 Giessen, Germany; Department of Radiology, University Hospital Giessen and Marburg, Justus Liebig University Giessen, Klinikstrasse 33, 35392 Giessen, Germany; Department of Cardiac and Vascular Surgery, University Hospital Giessen and Marburg, Justus Liebig University Giessen, Rudolph-Buchheim-Strasse 7, 35392 Giessen, Germany

**Keywords:** Sarcopenic obesity, GDF-15, Epicardial fat, TAVR, BMI

## Abstract

**Aims:**

To analyse the relevance of body composition and blood markers for long-term outcomes in very old patients after transcatheter aortic valve replacement (TAVR).

**Methods and results:**

A total of 403 very old patients were characterized with regard to subcutaneous, visceral, and epicardial fat, psoas muscle area, plasma growth differentiation factor 15 (GDF-15), and leptin. Cohorts grouped by body mass index (BMI) were analysed for long-term outcomes. Patients underwent transapical and transfemoral TAVR (similar 30-day/1-year survival). Body mass index >35 kg/m^2^ showed increased 2- and 3-year mortality compared with BMI 25–34.9 kg/m^2^ but not compared with BMI <25 kg/m^2^. Fat areas correlated positively to BMI (epicardial: *R*^2^ = 0.05, *P* < 0.01; visceral: *R*^2^ = 0.20, *P* < 0.001; subcutaneous: *R*^2^ = 0.13, *P* < 0.001). Increased epicardial or visceral but not subcutaneous fat area resulted in higher long-term mortality. Patients with high BMI (1781.3 mm^2^ ± 75.8, *P* < 0.05) and lean patients (1729.4 ± 52.8, *P* < 0.01) showed lower psoas muscle area compared with those with mildly elevated BMI (2055.2 ± 91.7). Reduced psoas muscle area and increased visceral fat and epicardial fat areas were independent predictors of long-term mortality. The levels of serum GDF-15 were the highest in BMI >40 kg/m^2^ (2793.5 pg/mL ± 123.2) vs. BMI <25 kg/m^2^ (2017.6 pg/mL ±130.8), BMI 25–30 kg/m^2^ (1881.8 pg/mL ±127.4), or BMI 30–35 kg/m^2^ (2054.2 pg/mL ±124.1, all *P* < 0.05). Increased GDF-15 level predicted mortality (2587 pg/mL, area under the receiver operating characteristic curve 0.94). Serum leptin level increased with BMI without predictive value for long-term mortality.

**Conclusion:**

Morbidly visceral and epicardial fat accumulation, reduction in muscle area, and GDF-15 increase are strong predictors of adverse outcomes in very old patients post-TAVR.

## Graphical Abstract

**Graphical Abstract:**
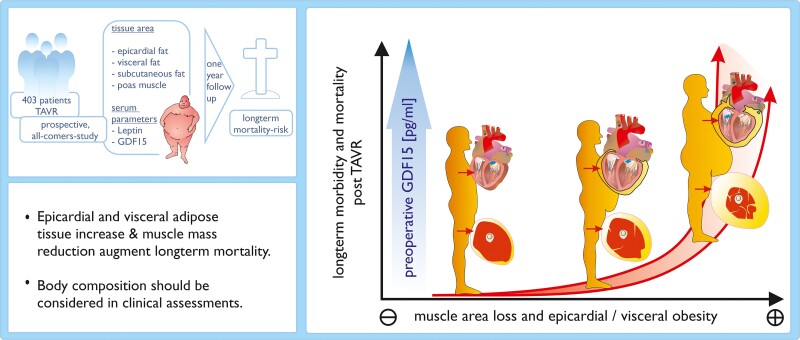

**Translational perspective**

Our data make it possible to identify high-risk patients in whom obesity and sarcopenia may increase post-procedural mortality even at very old age. This may allow applying secondary prophylactic measures after transcatheter aortic valve replacement in these patients.

## Introduction

Obesity is an established risk factor for cardiovascular diseases, Type 2 diabetes mellitus, cancer, or sleep apnoea, and is associated with a high burden of morbidity and mortality. Nevertheless, observational studies have shown that overweight or moderate obesity is associated with better outcomes in older patients with chronic diseases, a phenomenon termed ‘obesity paradox’.^1^ However, it has also been debated that the adjustment for potential confounders such as age, disease stage, smoking habit, or the course of weight changes may significantly scale down the protective effect of obesity.^2^ In fact, mortality seems to be the greatest in cachectic patients, lower in normal, overweight, and mildly obese individuals, but the highest in those with Grade IV obesity, suggesting a ‘U-shaped’ outcome curve according to body mass index (BMI).^1^

Different single-centre studies have shown that obesity and overweight are independently associated with better outcomes, supporting the obesity paradox in patients with transcatheter aortic valve replacement (TAVR).^3–5^ A meta-analysis encompassing 16 studies with more than 12 000 patients with TAVR showed protective effects of higher BMI and obesity on short- and long-term survival,^6^ which is also supported by another meta-analysis as well as recent registry data.^5,7^

In addition to the general measure of obesity such as BMI or waist circumference, the distribution of adipose tissue (AT) is important to understand the impact of site-specific fat depots on the whole organism. While subcutaneous AT (SAT) appears to be protective against an adverse prognosis, visceral AT (VAT) is associated with elevated levels of inflammatory mediators.^8^ Among these, the adipocyte-derived hormone leptin is a pro-inflammatory adipocytokine that is primarily expressed and secreted by visceral adipose tissue. A direct pro-inflammatory and metabolic modulating effect on the heart is feasible in a paracrine manner due to the absence of a muscle fascia between the epicardium and the myocardium. Furthermore, growth differentiation factor 15 (GDF-15) is a valuable predictor of poor outcome after TAVR^9,10^ and a biomarker of muscle weakness and low physical performance. It correlates with frailty independent of LV function in older patients with cardiometabolic risk.^11,12^

Epicardial AT (EAT), a visceral fat located between the myocardium and the pericardium, is biochemically different from intra-abdominal VAT, although they share a common embryological origin.^13,14^ A large EAT volume is associated with unfavourable endocrine activity and correlates with an increased cardiovascular risk.^13,15^

Besides obesity, patients undergoing TAVR are often individuals with reduced cardiorespiratory fitness and a reduced lean body mass. The term ‘sarcopenic obesity’ has been proposed to identify obesity with low skeletal muscle function and mass.^8,16^ Both sarcopenia and obesity promote adverse outcomes, although sarcopenic obesity may not confer a greater risk on patients than sarcopenia alone.^16,17^ In fact, weight loss, combined with sarcopenia, appears to present the greatest mortality risk.^17^ Various, mostly single-centre studies have revealed that lower psoas muscle area (PMA) or volume as an objective frailty assessment tool is an independent predictor of mortality after TAVR.^18–22^

Thus, in addition to general parameters of obesity, AT distribution, together with skeletal muscle area in a pre-operative, electrocardiogram (ECG)-gated, three-dimensional cardiovascular computed tomography (CT) scan, may help to predict morbidity and mortality in patients with TAVR. However, these data are currently calculated only individually and independently of each other, and are not included in risk score calculation, nor do they determine the clinical decision. The purpose of our present investigation is to analyse the impact of EAT, VAT, and SAT in context with changes in PMA and blood parameters on short- and long-term mortality in elderly patients undergoing TAVR. We hypothesize that increased amounts of fat tissue and sarcopenia heighten the risk for long-term mortality even in the elderly.

## Methods

### Patients

We included all patients from January 2014 to December 2017 undergoing TAVR implantation at our institution. Telephone interviews were performed at 30 days and 1, 2, and 3 years post-TAVR. Aortic valve disease was assessed initially with transthoracic echocardiography, followed by an ECG-gated, three-dimensional cardiovascular CT scan. Further details can be found in Supplementary material. Patients were assigned to sub-cohorts according to their BMI: lean (18.5 kg/m^2^ < BMI <25 kg/m^2^), mild overweight (25 kg/m^2^ < BMI <30 kg/m^2^), and Grade I obesity (30 kg/m^2^ < BMI < 35 kg/m^2^). Patients with Grade II (35 kg/m^2^ < BMI < 40 kg/m^2^) and Grade III (BMI > 40 kg/m^2^) obesity were grouped together (BMI > 35 kg/m^2^) due to the low patient number. None of our patients had a BMI <18.5 (underweight). The interventional risk was estimated using EuroSCORE II.

### Computed tomography scans and measurement of fat tissue area and psoas muscle area

All patients underwent a routine pre-procedural CT scan, for which a 128-slice or a 384-slice dual-source scanner was used (Somatom, Siemens Medical Solutions, Forchheim, Germany). A retrospective ECG-gated acquisition protocol was utilized for all measurements. The first level was set to the heart and a second, non-gated acquisition level to the remaining aorta and iliofemoral arteries. Contrast enhancement was obtained by a body-weight-adjusted application of 80–120 mL iodinated contrast medium through an antecubital vein (4 mL/s, afterwards, a bolus of 50 mL isotonic saline). The scans were timed by ECG peak enhancement detection within the ascending aorta, which was the region of interest. Reconstructions were carried out using a cardiac-gated B26f or I26f algorithm; thus, data were reconstructed in end-systole and end-diastole (30–40 and 60–70% of the R–R interval). Each measurement was individually quantified in standardized slices in terms of subcutaneous fat tissue area and diameter, visceral fat tissue area, PMA (level of the superior mesenteric artery), and epicardial fat tissue area and diameter (mid-papillary level) using Synedra view software (version 18.0.0.16(x64Edition) synedra AIM 18 ‘Apollon’. Individual measurement of fat tissue area was performed by manual exclusion of the area of all other thoracic or abdominal organs, avoiding automatic analysis. We normalized areas thereafter to body surface area (BSA), which was calculated by following Dubois [BSA (m^2^) = 0.007184×height (cm), 0.725×body weight (kg), 0.425]. All measurements were performed by an independent, trained observer who was blinded to clinical data.

### Indication for transcatheter aortic valve replacement, valves implanted, and baseline parameters

All patients suffered from severe symptomatic aortic stenosis and were assessed by an interdisciplinary heart team consisting of at least one cardiac surgeon, one cardiologist, and one cardiac anaesthesiologist. All patients underwent pre-procedural coronary angiography to assess the need for revascularization. Patients with a life expectancy of more than 1 year but not eligible for surgical valve procedures due to increased morbidity and peri-operative risk underwent TAVR. A transfemoral (TF) or transapical (TA) access approach was chosen on the basis of the individual patient’s risk profile. A variety of self-expanding and balloon expandable valves were implanted, ensuring the optimal choice for the individual patient. For TF access, we routinely used the Sapien™ XT (Edwards Lifesciences, Irvine, CA, USA), the self-expandable Acurate TA™ (Symetis, Ecublens, Switzerland), and the self-expandable CoreValve™ (Medtronic, Minneapolis, MN, USA). For TA access, the most commonly used valves were the balloon-expandable Sapien™ XT (Edwards Lifesciences) and the self-expandable Acurate TA™ (Symetis). We performed 30-day, 1-year, 2-year, and 3-year follow-ups for all patients with all-cause mortality as the primary endpoint. Baseline clinical characteristics included age, sex, height, weight, BMI, BSA, serum C-reactive protein, lactate, leucocytes, and estimated glomerular filtration rate. In addition, we analysed peri-operative morbidity and inflammatory activation.

### Enzyme-linked immunosorbent assay

Plasma GDF-15 and leptin were measured in triplicate using commercial enzyme-linked immunosorbent assays (Human GDF-15 ELISA, RayBiotech; Human Leptin ELISA, RayBiotech) according to the manufacturer’s instructions.

### Ethics vote and implementation of the study

The institutional ethics committee approved the study protocol (AZ 196/17) as an observational study. Informed patient consent was obtained prior to observation. The study was conducted in accordance with national and institutional guidelines and laws (Section 53 Hess. Higher Education Act; Directive 2001/20/EC of the European Parliament and of the Council of 4 April 2001; German Federal Law Gazette year 2004, Part I, No. 42) by following the principles outlined in the Declaration of Helsinki.

### Statistical analyses

Continuous variables are expressed as mean ± standard deviation, in case of Gaussian variable distribution, otherwise as median and 25%/75% confidence intervals (CIs), as indicated. Categorical data are expressed as relative units of the total. Comparisons of nominal variables were performed using a *χ*^2^ test or Fisher’s exact test. Comparisons of two groups were performed by using a *t*-test; differences among many groups were analysed by one-way analysis of variance (ANOVA). Differences in mortality were examined with Kaplan–Meier estimates. Univariate and multivariate multiple regression analyses were performed to calculate odds ratios. A receiver operating characteristic (ROC) curve analysis was performed by plotting sensitivity on the *y*-axis as a function of one specificity on the *x*-axis to analyse the diagnostic ability of plasma GDF-15 in terms of mortality. Statistical significance was defined as *P* < 0.05 and beta > 0.8. Statistical analysis was performed using SigmaStat for Windows® V3.5.0.54 (Systat Software Inc.). The data were collected and analysed using IBM SPSS Statistics version 27 (IBM Corp., USA).

## Results

### Characteristics of patients

Overall, 403 mainly very old patients (mean age 81.5 ± 6.1 years; 51% female) with symptomatic severe aortic stenosis undergoing TAVR were included in our analysis. Among this cohort, 152 patients had normal weight (37.7%), 158 patients were mildly overweight (39.2%), 60 patients belonged to the Grade I obesity group (14.9%), and 33 patients belonged to the Grade II + III obesity group (8.2%). Among lean patients, the lowest BMI was 21 kg/m^2^. Details are given in *Table 1* and within the Supplementary material regarding the characteristics of patients and selected inflammatory, metabolic, and renal serum parameters (Supplementary material online, *Figure S2*).

**Table 1:** Baseline characteristics of patients with transcatheter aortic valve replacement

	All TAVR patients	Transfemoral access (TF)			Transapical access (TA)			*P*-value TF vs. TA
		Median	25%	75%	Median	25%	75%	
*n*	403	336			67			
Age (years)	81.3 ± 6.6	81.5	78.1	84.9	81.5	77.5	84.9	0.76
Male gender [*n* (%)] #	197 (48.9)	159 (47.3)			38 (56.7)			1
BSA (Dubois, m^2^)	1.85 ± 0.209	1.86	1.701	1.996	1.81	1.688	1.962	0.12
BMI (kg/m^2^)	27.2 ± 5.0	26.6	23.7	30.1	25.5	23.1	28.3	<0.01
BMI < 25 [*n* (%)] #	152 (37.7)	120 (35.7)			32 (47.8)			0.04
25 < BMI < 30 [*n* (%)] #	158 (39.2)	134 (39.9)			24 (35.8)			0.98
30 < BMI < 35 [*n* (%)] #	60 (14.9)	52 (15.5)			8 (11.9)			0.32
BMI > 35 [*n* (%)] #	33 (8.2)	30 (8.9)			3 (4.5)			0.20
1-Vessel CAD [*n* (%)] #	66 (16.4)	55 (16.4)			11 (16.4)			0.86
2-Vessel CAD [*n* (%)] #	78 (19.4)	63 (18.8)			15 (22.4)			0.60
3-Vessel CAD [*n* (%)] #	104 (25.8)	74 (22.0)			30 (44.7)			<0.001
Left main stem CAD [*n* (%)] #	26 (6.5)	10 (14.9)			16 (23.9)			<0.001
NYHA I [*n* (%)] #	2 (0.4)	2 (0.6)			0 (0)			0.37
NYHA II [*n* (%)] #	114 (28.4)	95 (28.3)			19 (28.4)			0.96
NYHA III [*n*(%)] #	229 (56.8)	196 (58.3)			33 (49.2)			0.46
NYHA IV [*n* (%)] #	58 (14.4)	43 (12.8)			15 (22.4)			0.17
LVEF (%)	55.2 ± 11.7	60	50	65	60	50	65	0.78
EuroSCORE II (%)	6.63 ± 7.1	4.32	2.12	7.49	5.40	2.55	11.02	0.12
Arterial hypertension [*n* (%)] #	393 (97.5)	327 (97.3)			66 (98.5)			0.89
Pulmonary hypertension [*n* (%)] #	197 (48.9)	164 (48.8)			33 (49.3)			0.95
Hypolipoproteinaemia [*n* (%)] #	294 (72.9)	239 (71.1)			55 (82.1)			0.09
Diabetes mellitus [*n* (%)] #	145 (36.0)	120 (35.7)			25 (37.3)			0.91
Stroke [*n* (%)] #	57 (14.1)	45 (13.4)			12 (17.9)			0.44
PAD/carotid AD [*n* (%)] #	110 (27.3)	74 (22.0)			36 (53.7)			<0.001
Pneumonia [*n* (%)] #	37 (9.2)	32 (9.5)			5 (7.5)			0.76
COPD [*n* (%)] #	80 (19.9)	62 (18.5)			18 (26.9)			0.16
Lung fibrosis [*n* (%)] #	7 (1.7)	7 (2.1)			0 (0)			0.50
Prior cardiac surgery [*n* (%)] #	110 (27.3)	93 (27.7)			17 (25.4)			0.81
Effective orifice area (cm^2^)	0.8 ± 0.2	0.8	0.6	0.9	0.75	0.7	0.9	0.87
Aortic valve gradient (mmHg)	37.5 ± 16	36	25	47	35	25	44	0.33
Glomerular filtration rate (mL/min)	66.7 ± 29.2	66.8	49.8	85.8	55.8	35	75.2	<0.01
Leucocytes (10^9^/L)	8 ± 3.6	7.3	6	9	7.8	6.5	9	0.28
Lactate (mg/dL)	1.3 ± 0.8	1.1	0.7	1.7	0.9	0.7	1.4	0.09
Creatinine (mg/dL)	1.6 ± 5.2	1	0.8	1.4	1.2	0.9	1.7	0.08
Urea (mg/dL)	55.5 ± 32.5	46	34	64	50	39	72	0.11

*P*-values for the comparison of transapical vs. transfemoral access. Continuous variables are expressed as mean ± standard deviation (Gaussian variable distribution; indicated with #), otherwise as median and 25%/75% confidence intervals. Categorical data are expressed as *n* and relative units of the total. Comparisons of nominal variables were performed using *χ*^2^ test or Fisher’s exact test. Comparisons of two groups were performed by *t*-test; differences between many groups were analysed by one-way ANOVA. Statistical significance was defined as *P* < 0.05 and beta > 0.8.

BMI, body mass index; BSA, body surface area; CAD, coronary artery disease; carotid AD, carotid artery disease; PAD, peripheral artery disease.

### Peri-operative course, survival, and morbidity

**Figure 1:**
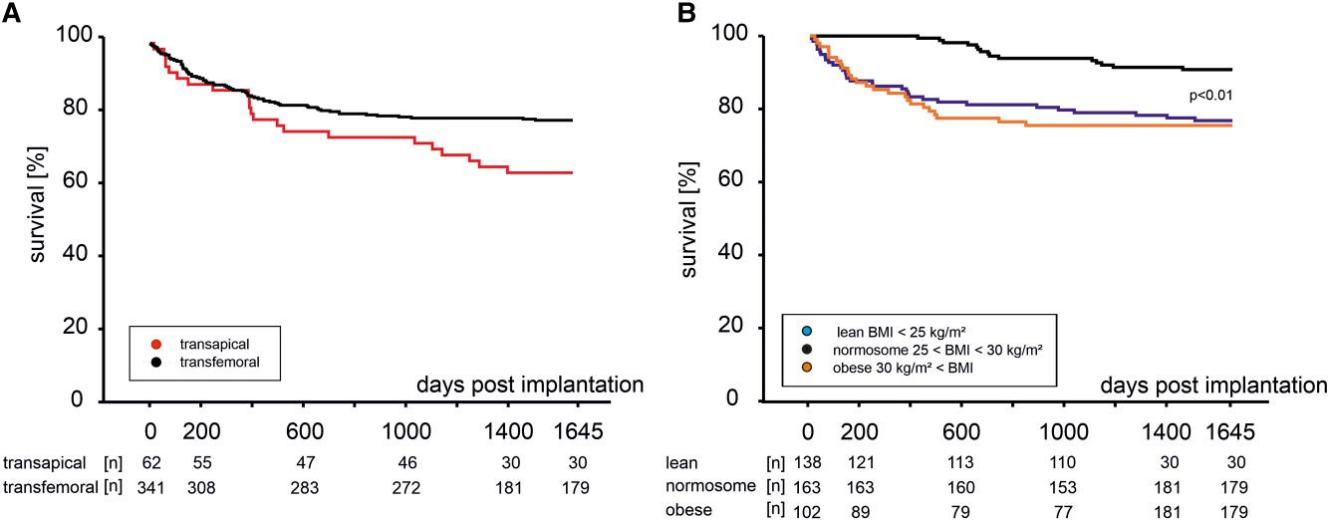
Kaplan–Meier survival curve of patients with severe aortic stenosis undergoing transcatheter aortic valve replacement. (*A*) Stratified according to access approach and (*B*) stratified according to body mass index up to 1645 days post transcatheter aortic valve replacement.

**Figure 2:**
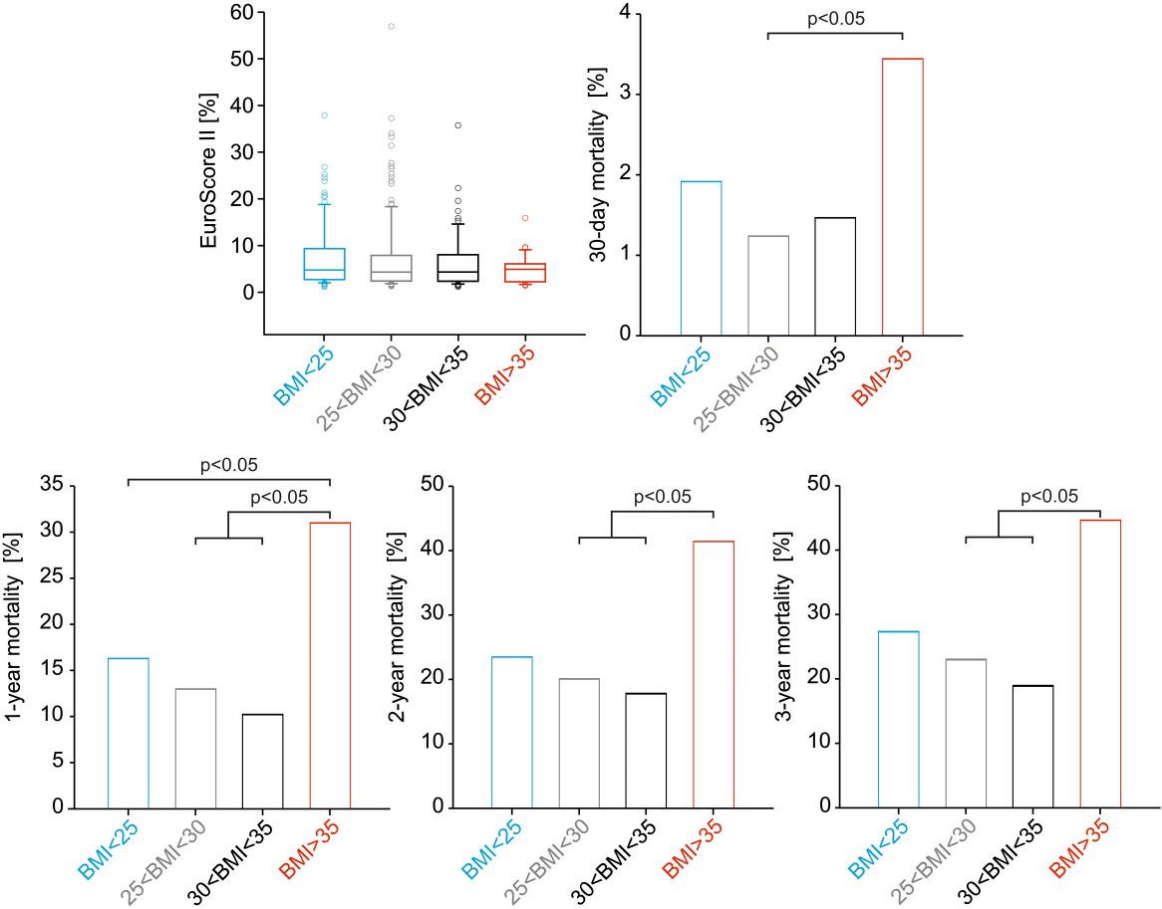
Predicted and actual mortality. Risk calculation by EuroSCORE II and post-operative 30-day and long-term outcomes (1, 2, and 3 years) according to body mass index. Assigned subgroups: lean (body mass index < 25 kg/m^2^, *n* = 152), mild overweight (25 kg/m^2^ < body mass index < 30 kg/m^2^, *n* = 158), and Grade I obesity (30 kg/m^2^ < body mass index < 35 kg/m^2^, *n* = 60), and Grades II and III obesity (body mass index > 35 kg/m^2^, *n* = 33). None of the patients had a body mass index < 18.5. Data were given as mean ± standard deviation or as median and 25%/75% confidence intervals if not normally distributed. The data were analysed by using one-way analysis of variance followed by Tukey’s test.

There was no significant difference in cumulative mortality rates up to 1645 days after TAVR between patients with TA and TF (*Figure 1A*), with a similar mean survival (30 days/1 year) for patients with TA (97/86%) and TF (98/87%). A cumulative mortality analysis of all patients undergoing TAVR demonstrated an increased mortality in BMI > 30 kg/m^2^ compared with mildly overweight patients (25 < BMI < 30 kg/m^2^). Patients with obesity showed an early-onset and long-term constant increased mortality (Kaplan–Meier analysis, *Figure 1B*). In terms of selection criteria, all patients with TAVR pre-operatively showed chronic disease courses and an increased EuroSCORE II mortality risk. As frailty or obesity has not been incorporated in EuroSCORE II or the Society of Thoracic Surgeons, there was no difference in the EuroSCORE II-predicted risk of death among the different BMI groups (*Figure 2*). While the calculation for lower body weights coincides with this prediction, we observed a significant increase in 30-day mortality in Grade II or Grade III obesity (BMI > 35 kg/m^2^; *Figure 2*). Furthermore, this effect is maintained and further increased 1, 2, or 3 years after TAVR (*Figure 2*). The lowest long-term mortality rates were observed in patients with Grade I obesity (*Figure 2*).

The Supplementary material and Supplementary material online, *Figure S1* gives detailed data on operative procedures and peri-operative course. Successful device delivery rate and device implantation was equal between subgroups. Conversion to surgical aortic valve replacement (SAVR) was necessary in a small sub-cohort of lean (1.9%), mildly obese patients (1.9%) and in 5.7% of patients with morbid obesity (*P* = NS, Supplementary material online, *Figure S1A*). No difference with regard to surgical revisions, rates of permanent pacemaker implantation, procoagulatory medications, or permanent neurological deficits existed between the BMI groups (see Supplementary material online, *Figure S1A*).

### Adipose tissue and muscle area

**Figure 3:**
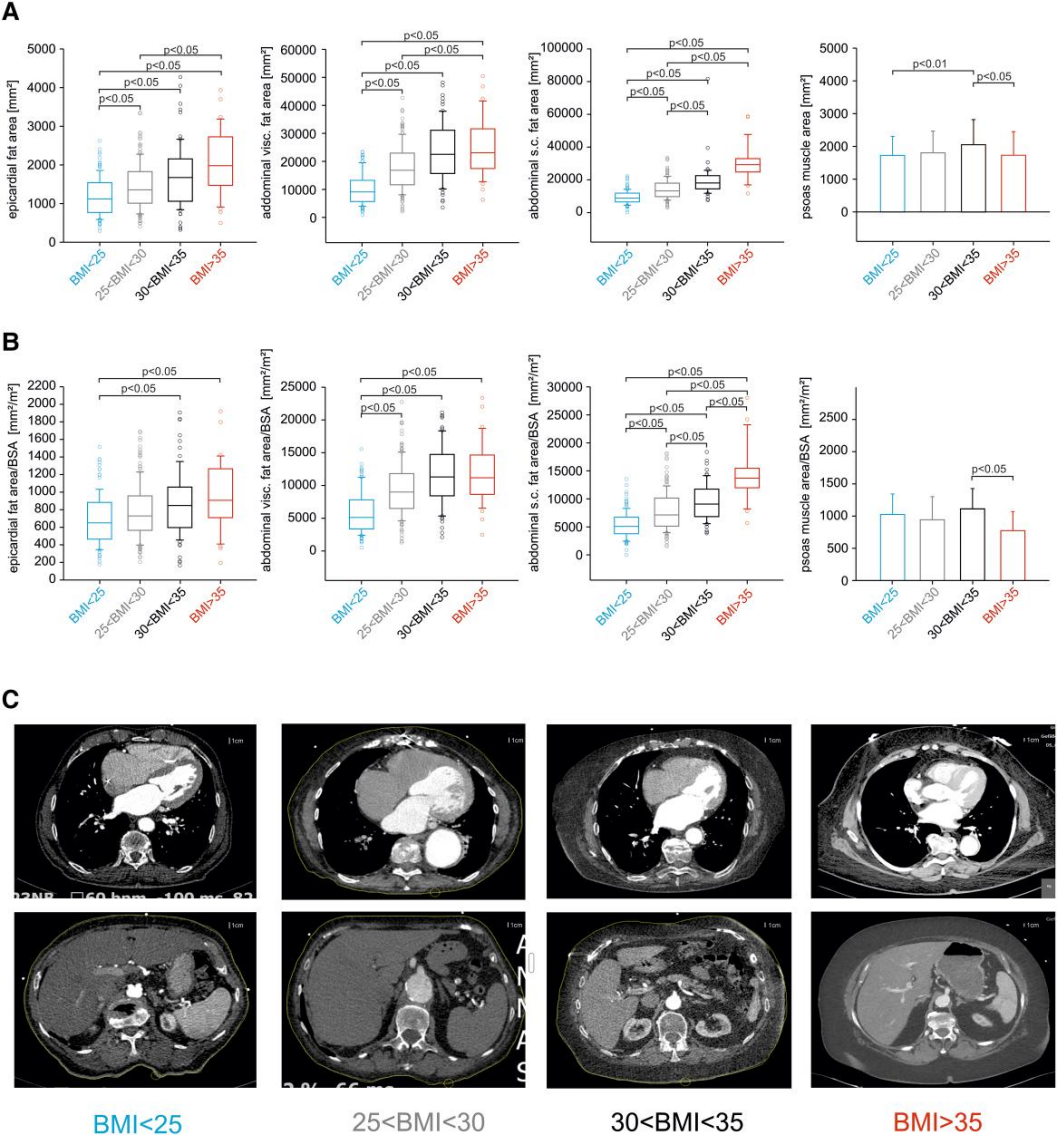
Adipose tissue and psoas muscle area. (*A*) Epicardial, abdominal visceral (visc.), abdominal subcutaneous (s.c.) fat area and psoas muscle area (mm^2^). (*B*) Areas according to *A* normalized to body surface area in mm^2^/m^2^. Groups as described in *Figure 2*. Data given as mean ± standard deviation or as median and 25%/75% confidence intervals if not normally distributed. The data were analysed by one-way analysis of variance followed by Tukey’s test. (*C*) Representative pre-procedural computed tomography scans to demonstrate qualitative distribution of tissues. Each picture: complete body diameter. Scale bar: 1 cm/individual patient.

Body mass index correlates with increasing EAT, abdominal VAT, and abdominal SAT (*Figure 3*) before and after normalization to BSA. Thus, BMI and BSA were associated with normalized EAT, VAT, or SAT area (see Supplementary material online, *Figure S3A*). Psoas muscle area, however, was the highest in patients with Grade I obesity (30 kg/m^2^ < BMI < 35 kg/m^2^) compared with patients with lower or higher BMI (*Figure 3A*). After normalization to BSA, a significantly reduced muscle area was detected in patients with severe obesity (BMI >35 kg/m^2^) compared with patients with Grade I obesity (*Figure 3B*). Unlike AT, no significant association was detectable between BMI and PMA (see Supplementary material online, *Figure S3B*). While BSA showed a strong positive correlation with BMI, increasing age showed a weak inverse association with BMI (see Supplementary material online, *Figure S3B*). In addition, no association was observed between EAT, VAT, or SAT and PMA (see Supplementary material online, *Figure S3C*).

**Figure 4:**
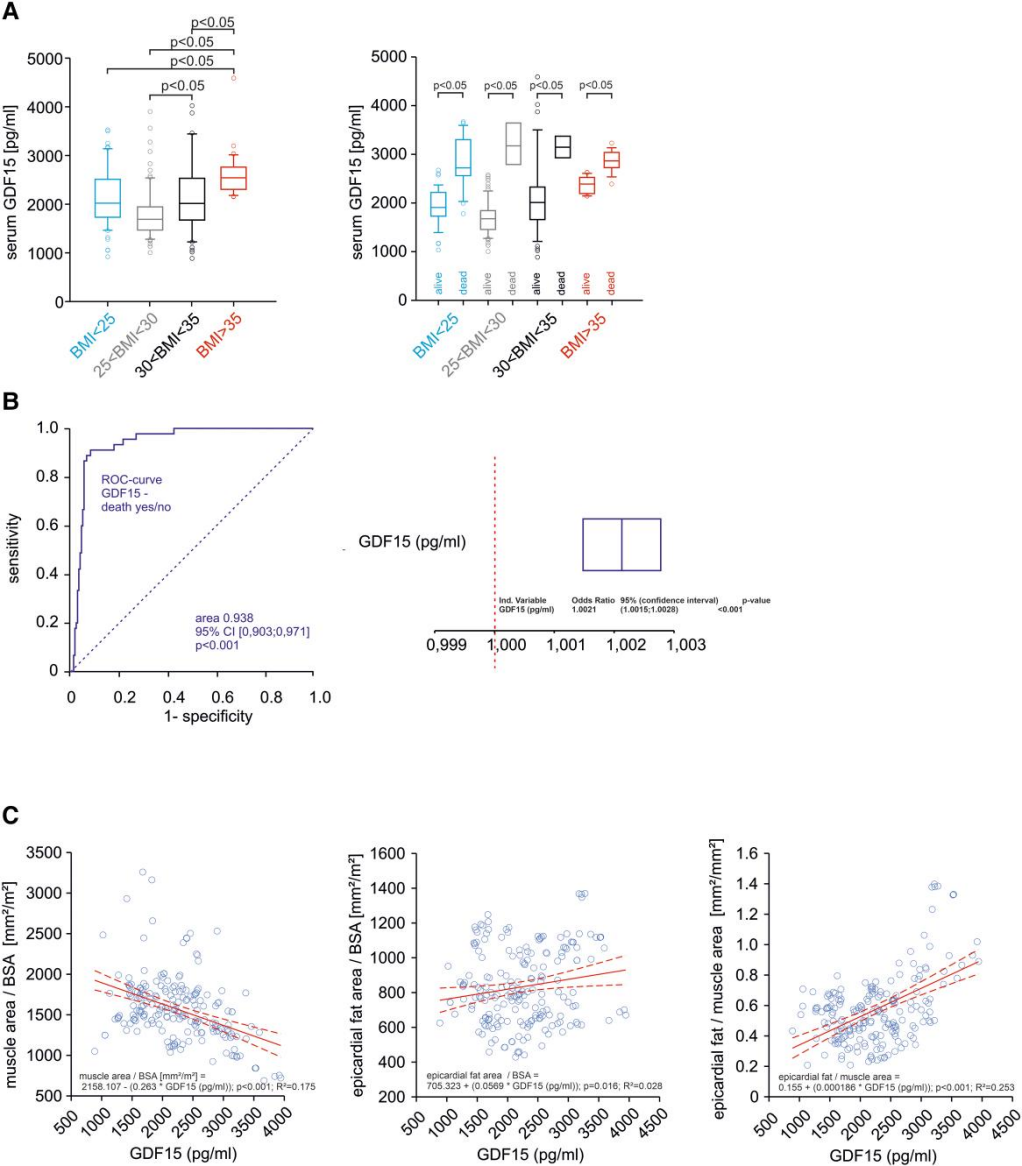
Serum growth differentiation factor 15. (*A*) Pre-operative serum growth differentiation factor- 15 in all patients according to the body mass index groups or divided into surviving and deceased patients. Groups as described in *Figure 2*. Data given as median and 25%/75% confidence intervals and were analysed by one-way analysis of variance followed by Tukey’s test. (*B*) Receiver operating characteristic curve for serum growth differentiation factor 15 for the prediction of long-term mortality (left panel). Univariate analysis of effects of growth differentiation factor 15 serum concentration increase for 1-year mortality (right panel). (*C*) Regression analyses of serum growth differentiation factor 15 and body surface area–normalized muscle area, body surface area–normalized epicardial fat area, or epicardial fat/muscle area. Data from 403 patients with transcatheter aortic valve replacement.

Fat and muscle area and cytokine serum concentrations were examined with regard to their predictive value as predictors of 1-year mortality in a univariate regression analysis (*Table 2A*). We normalized measurements of fat tissue areas to body surface. Body mass index, VAT, and EAT as well as PMA reduction and increased GDF-15 serum concentration were predictors of 1-year mortality (univariate analysis). All despite BMI were also predictors in the multivariate analysis (*Table 2B*). A significant difference in abdominal fat area/BSA but not EAT/BSA was observed when comparing surviving patients and patients deceased within 1 year (see Supplementary material online, *Figure S4*). The question whether vascular complications or stroke were related to EAT (not shown) or VAT was also analysed (see Supplementary material online, *Figure S4*). The total heart area increased with BMI (see Supplementary material online, *Figure S5*) as a result of EAT, not cardiac hypertrophy [interventricular septum diameter in diastole (IVSDd); left ventricular end diastolic diameter (LVEDD), Supplementary material online, *Figure S5*]. Accordingly, BSA-normalized cardiac area was not different between the BMI groups (see Supplementary material online, *Figure S5*). The ROC analysis for epicardial, subcutaneous, and visceral fat tissue area and PMA for 1-year mortality is given in *Figure 4*. Furthermore, 1-year mortality is predictable by increase in epicardial adipose tissue, visceral fat area/total abdominal area, and total AT area. Increased PMA predicts better survival (all data given in *Figure 4*). The threshold value (Youden index) of the PMA is 2627.00 mm^2^ and epicardial fat area is 1671.94 mm^2^, the ratio of visceral fat area/abdomen area is 0.31, and the total fat area is 28 755.90 mm^2^.

**Table 2:** Univariate (A) and multivariate (B) logistic regression analysis

A	Univariate analysis	Regression coefficient	Standard deviation	Odds ratio	95% Confidence interval for OR		Significance (*P*-value)
					5% lower bound	95% upper bound	
	BMI (kg/m^2^)*	0.059	0.023	1.061	1.014	1.111	0.011
	GDF-15 (pg/mL)*	0.002	0.000	1.002	1.001	1.003	0.000
	Leptin (pg/mL)	0.000	0.000	1.000	10.00	1.000	0.292
	Epicardial fat tissue area (mm^2^)*	0.001	0.000	1.001	1.001	1.002	0.000
	Subcutaneous fat tissue area (mm^2^)	0.000	0.000	1.000	1.000	1.000	0.186
	Visceral fat tissue area (mm^2^)*	0.000	0.000	1.000	1.000	1.000	0.000
	Psoas muscle area (mm^2^)*	−0.004	0.001	0.996	0.995	0.997	0.000

B	Multivariate analysis				95% Confidence interval for OR		
		Regression coefficient	Standard deviation	Odds ratio	5% Lower bound	95% Upper bound	Significance (*P*-value)
	BMI (kg/m^2^)	−0.225	0.130	0.799	0.619	1.030	0.084
	GDF-15 (pg/mL)	0.004	0.001	1.004	1.002	1.007	0.001
	Epicardial fat tissue area (mm^2^)	0.002	0.001	1.002	1.001	1.003	0.001
	Visceral fat tissue area (mm^2^)	0.000	0.000	1.000	1.000	1.001	0.007
	Psoas muscle area (mm^2^)	−0.009	0.003	0.991	0.986	0.996	0.001

Covariates used: BMI (kg/m^2^), serum concentration of cytokines (GDF-15; Leptin), fat tissue planimetry, and muscle planimetry. Single measurement for each variable per patient (*n* = 403) for planimetries. Mean of three independent ELISA measurements for GDF-15 and leptin. Significance was assumed at a *P* < 0.05. Asterisks indicate the significant variables in univariate analysis in (A) used for further multivariate analysis in (B). BMI, body mass index (kg/m^2^); GDF-15, growth differentiation factor 15.

### Serum growth differentiation factor 15 and leptin levels in very old patients, obesity, and muscle loss

**Figure 5:**
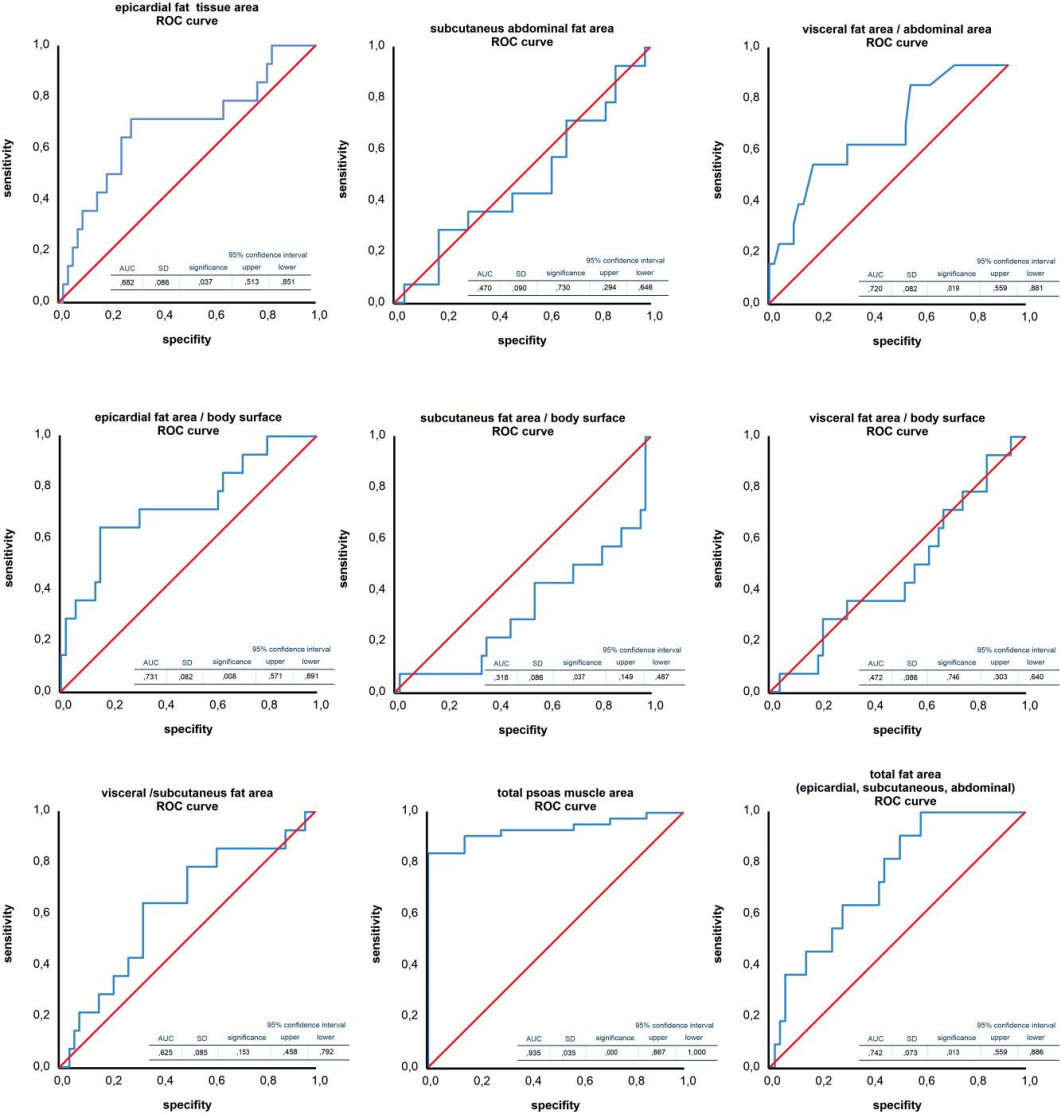
Receiver operating characteristic curve analyses of the analysed areas. First row: epicardial, subcutaneous, and visceral fat areas. Second row: indicated areas epicardial, subcutaneous on body surface, and visceral fat area on abdominal area. Third row: visceral/subcutaneous fat area, psoas muscle area, and total fat area.

In the present study, serum GDF-15 was significantly increased in severely increased BMI > 35 kg/m^2^, suggesting impaired skeletal muscle performance in these patients (*Figure 5A*). Long-term mortality was associated with higher GDF-15 serum levels in all groups, emphasizing its importance as a biomarker (*Figure 5A*). The ROC curve analyses showed a cut-off value for GDF-15 of 2587 pg/mL [area under the ROC curve (AUC) 0.94, 95% CI 0.90–0.97, *P* < 0.001, sensitivity 91.0%, and specificity 91.0%] for mortality (*Figure 5B*). A 1 pg/mL GDF-15 increase results in an increase of 0.21% of 1-year mortality (*Figure 5B*). Regression analyses showed that a high GDF-15 serum level is a strong predictor of low PMA and a weaker predictor of high EAT (*Figure 5C*). This effect of GDF-15 is consistent with significantly lower PMA in patients with severe obesity (*Figure 3*). Serum leptin increased with BMI and showed the highest levels in severe obesity (see Supplementary material online, *Figure S6*). It was strongly correlated to BMI in all patients (Pearson correlation coefficient *r* = 0.68, *P* < 0.001) but was not a predictor of increased mortality.

## Discussion

The results of this study suggest that patients with severe obesity undergoing TAVR show the highest short- and long-term mortality despite similar interventional procedures, cardiac hypertrophy, surgical results, or peri-interventional course. This subgroup of patients demonstrates the highest amount of VAT, SAT, and EAT but lowest PMA. In addition, serum GDF-15, a previously reported predictor of a poor outcome after TAVR,^9,21^ is the highest in this subgroup of patients with TAVR. In our study, reduced PMA is a strong, independent predictor of short and long-term mortality, while VAT and EAT areas are strong, independent predictors of long-term mortality. We collected data from a cohort of chronically ill and very old patients. Interestingly, the harmful effects of obesity and PMA reduction gained relevance in the medium term after surgery. Indeed, the impact of access and overweight/obesity on mortality demonstrates a different time course. Therefore, we illustrate this difference in *Figure 1A* and *B*. Patients with TA and TF differ in three vessel coronary artery disease (CAD), main stem stenosis, renal function, and carotid artery disease, as shown in *Table 1*. These differences may explain increased long-term mortality in patients with TA. However, obesity groups did not differ regarding three vessel CAD, main stem stenosis, renal function, and carotid artery disease but exhibited increased mortality in the short term. Thus, obesity appears to be an independent confounder of mortality in patients with TF and TA. Apart from TAVR, obesity and loss of muscle area could also influence outcomes in other cohorts of very old patients and supplement the assessment of frailty and associated morbidity in the elderly.

### Obesity paradox

Observational studies show that overweight or moderate obesity is associated with better outcomes in older patients with chronic diseases, leading to the medical hypothesis of an ‘obesity paradox’.^1^ However, these studies have been criticized for limitations, such as the use of BMI and various types of bias.^2^ In addition, the adjustment for potential confounders, such as age, disease stage, smoking habit, or the course of weight changes, may significantly scale down the protective effect of obesity on mortality.^2^ Interestingly, in a large, pooled analysis, both overweight and obesity (and possibly underweight) were associated with increased all-cause mortality in an analyses restricted to healthy participants who never smoked.^23^ In these pooled data from 19 prospective studies encompassing 1.46 million white adults between the ages of 19 and 84 years, the lowest all-cause mortality was generally observed in the BMI range of 20.0–24.9 after adjustment for age, study, physical activity, alcohol consumption, education, and marital status.^23^ Our study shows the highest mortality in patients with Grade II or III obesity (BMI > 35 kg/m^2^) within 30 days as well as 1, 2, or 3 years after TAVR. The lowest long-term mortality rates were observed in patients with overweight or Grade I obesity (25 kg/m^2^ < BMI < 35 kg/m^2^). Other TAVR studies also reported better acute outcomes and long-term survival in patients with obesity or overweight.^3–7^ Unlike in our study, a detailed BMI subgroup analysis is not reported in most of these studies and limited data regarding outcomes in very obese patients exist. A recent registry analysis of nearly 32 000 patients reported the highest 30-day and 1-year mortality in underweight patients, lowest mortality in patients with a BMI around 30 kg/m^2^, and a gradual increase in mortality associated with severe obesity in patients with TAVR.^5^

### Psoas muscle area loss and obesity

Often, obesity is only referred to general measures, such as BMI or waist circumference,^24,25^ which neglect the distribution of solid tissues and fat tissues. In addition, the effects of obesity are much more complex in older individuals.^26^ Above the age of 65 years, the BMI does not differentiate between fat and muscle and does not integrate age-associated changes in body composition. Our patients with a BMI above 35 kg/m^2^ demonstrate not only the highest amount of VAT/SAT/EAT but also lowest PMA, suggesting a condition called sarcopenic obesity. Sarcopenic obesity is associated with higher morbidity and mortality and leads to frailty. Functional characterizations of muscle strength are lacking in our study to identify our patients as sarcopenic by definition. Nevertheless, with PMA and the biomarker GDF-15 validated for muscle weakness, we have a strong surrogate of sarcopenia. While the SAT appears to be protective against an adverse prognosis, the VAT appears to be associated with elevated levels of inflammatory meditators and worse metabolic profile.^8^ The VAT may thus directly contribute to progression of sarcopenia.^8^ Therefore, it has been suggested to distinguish between sarcopenic subcutaneous obesity and sarcopenic visceral obesity.^8^ This is in accordance with our data: an increase in EAT or VAT but not in SAT resulted in higher short- and long-term mortality.

### Effect of different fat depots

Aged patients are prone to adverse endocrine and inflammatory effects of VAT. A number of studies systematically investigated the influence of different fat compartments on the peri-procedural course and long-term outcomes of patients during and after TAVR.^4,27–30^ Increased EAT might be associated with a higher mortality.^28^ Others reported an association of lower SAT or VAT with an increase in adverse events.^27,29,30^ Most of these studies did not report outcome data sorted by different BMI subgroups. In obesity conditions a high VAT was associated with increased mortality, while in non-obese patients, an inverse association of VAT with mortality was observed.^4^ Different fat tissues originate from different embryologic sources and exhibit characteristic expressional and endocrine patterns.^14^ The present study suggests that in addition to VAT, EAT is an independent predictor of long-term mortality. Epicardial AT is not separated from the myocardium by a muscle fascia and shares its blood supply by branches of the coronary arteries.^13^ This allows a direct interaction and crosstalk between EAT and the myocardium via mediators such as cytokines or adipokines but also via cells. A large EAT volume is associated with unfavourable endocrine activity and was recognized as a predictor of adverse cardiac events including myocardial infarction or atrial fibrillation^15,31^ as well as increased mortality after TAVR.^28^ Other recently published single-centre studies showed that increased EAT volume or EAT density is predictors of increased mortality in patients undergoing TAVR.^32,33^ This is in accordance with the results of our study showing that EAT accumulation is a strong predictor of adverse outcomes in very old patients post-TAVR.

### Effect of psoas muscle area loss and frailty

In addition to increased AT, severe obesity also exhibited decreased PMA and increased serum GDF-15, as markers for muscle weakness.^11^ A comparison of different frailty scores and objective assessment of sarcopenia by imaging techniques revealed that normalized PMA is a strong predictor of long-term mortality after TAVR.^19^ Reduced PMA as a frailty assessment tool was shown in various studies to be a valid predictor of early or late morbidity, mortality, use and calculation of medical resources after SAVR and TAVR.^18,20–22,34^ A minority indeed investigated body composition and reported pre-TAVR CT data on PMA and AT area.^27,35^ Mok *et al*.^35^ reported that AT had no association with clinical outcomes, but sarcopenia predicted cumulative mortality. Both decreased AT and decreased PMA were associated with long-term mortality after TAVR by another group.^27^ Unlike our investigations, the latter studies did not analyse the impact of epicardial AT on TAVR outcome. Similarly, these studies did not implement biomarkers of muscle area, mass, strength, or function.^36^ Higher plasma leptin has been associated with a greater risk of frailty in older adults, independently from insulin resistance or chronic inflammation.^37^ Serum GDF-15, conversely, has proven to be a strong predictor of long-term mortality after TAVR in our study and others.^9,10^ Importantly, GDF-15 represents a biomarker of muscle weakness and low physical performance and correlates with frailty.^11,12^ Mechanistically, it has been suggested that GDF-15 is a myokine produced and released into circulation by skeletal muscle in response to ageing-related diseases and mitochondrial dysfunction.^38^ Our study suggests that GDF-15 is not only increased in patients with low PMA but also with high EAT. It may therefore serve as a valuable biochemical marker for the morphological changes observed in patients with sarcopenic obesity. However, our data rely largely on PMA measurements as we did not assess muscle strength or other functional muscle parameters for sarcopenia evaluation. Future experimental studies should also investigate the mechanisms involved in obesity-induced GDF-15 release by muscle cells and how adipocytes may contribute to the GDF-15 release.

### Limitations, generalizability, and risks of bias

Due to the non-multicentric data collection, data from our study cannot be generalized without a corresponding multicentric examination. A selection bias could be minimized by the complete inclusion and the complete follow-up. The image analyses were always carried out by the identical examiner. This examiner was blinded with regard to the individual outcome through the prospective data collection and with regard to the group outcomes. Statements regarding the effect of sarcopenia cannot be made due to the lack of muscular-functional data, so the data related to the area variance of the muscles are a correlate of such a statement.

Through telephone interviews, we were able to obtain information about the survival of all patients from either the study participants personally or from their relatives. An attrition bias is therefore unlikely. Since all parameters were collected using identical methods for each patient, a detection bias is also unlikely. We had no influence on the subsequent therapies of the individual groups. A performance bias cannot therefore generally be ruled out. Obesity accelerates biological ageing; so that obese groups are biologically older than normal cohorts (see Supplementary material online, *Figure S7*). Therefore, an age-weighted selection bias cannot be generally ruled out, but since a decision to undergo TAVR was made for all patients based on the degree of disease and frailty and an all-comers collective was selected, the bias risk can be assessed as low.

An increased BMI may induce other comorbidities such as hyperlipidaemia, CAD, hypertension, obstructive sleep apnoea, and Type 2 diabetes that contribute to an increased morbidity in patients with TAVR. Our high BMI groups were slightly younger compared with their lean counterparts (see Supplementary material online, *Figure S7*). This can be interpreted as a sign that obesity leads to a premature onset of cardiac pathologies. However, our data could also lead to a discussion regarding the meaningfulness of exclusively using BMI as a prognostic indicator. In fact, it might make more sense to use body composition including fat distribution and skeletal muscle condition instead. Our results do not describe a negative result in the narrower sense, so we do not assume publication bias. Since all patients were characterized without symptoms at the time of the operation and therefore had the same time of assessment, it is also unlikely that lead time bias was the cause of the time of death. Due to the limitation to the follow-up observation period, an effect that comes into play after this period cannot be analysed in cohorts that may appear protected. In this respect, medium-term effective pathologies that lead to death are becoming clearer. These include obesity and sarcopenia. Thus, a length bias in the general characterization of causes of death cannot be ruled out, but these are not the subject of our investigation.

Although GDF-15 proved to be a valuable predictor in our study, Leptin and GDF-15 ELISA are currently widely available only in basic science. Unfortunately, clinical availability as a routine parameter is not yet established and is costly. An establishment should be tested prospectively in a large cohort based on data from small cohorts such as this one.

## Conclusions

Traditional surgical risk scores do not capture the altered risk of very obese and sarcopenic patients. Therefore, they could be accompanied by CT-derived AT characteristics and serum markers such as GDF-15 to allow a more precise assessment of mortality (*Graphical Abstract*). A combination of clinical parameters, imaging data, and circulating biomarkers can serve as a risk stratification tool to define the frailty phenotype and to identify patients at risk of increased morbidity and mortality after TAVR. Addressing these risk factors could further consolidate a therapeutic benefit in mid-term follow-up of the elderly.

## Lead author biography


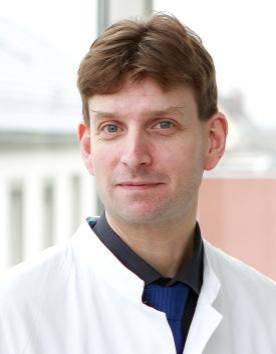


Prof. Dr Bernd Niemann 2002–10 trained as a cardiac surgeon at the University Hospital Halle and University Hospital Giessen and Marburg (UKGM-Gießen), a cardiac surgeon, specialist intensive care medicine, and a senior physician in cardiac surgery at the UKGM Gießen. His specializations were in the areas of heart failure (minimally invasive) heart valve, and coronary, aortic, and rhythm surgery. He obtained his doctorate on the endothelin system at Martin Luther University Halle/Wittenberg. He secured Venia legendi in cardiac surgery for metabolic cardiomyopathy.

## Supplementary Material

oeae073_Supplementary_Data

## Data Availability

The data underlying this article are available in the article and in its online Supplementary material.
