# Supplementary material for: Epicardial adipose tissue and muscle distribution affect outcomes in very old patients after transcatheter aortic valve replacement
Source: Eur Heart J Open. 2024 Sep 20;4(5):oeae073. doi: 10.1093/ehjopen/oeae073 (PMC11414403; doi:10.1093/ehjopen/oeae073)
Supplement: oeae073_Supplementary_Data [file oeae073_supplementary_data.zip › Supplement Revision 1.docx]

**Supplement to:**

**Epicardial adipose tissue and muscle distribution affect outcome in very old patients after TAVR**

**Susanne Rohrbach ^a^ *, Oezge Uluocak ^b^*, Marieke Junge ^b^, Fabienne Knapp ^a^,**

**Rainer Schulz ^a^, Andreas Böning ^b^, Holger M. Nef ^c^,**

**Gabriele A. Krombach ^d^, and Bernd Niemann ^b^**

*both authors contributed equally

^a^ Institute of Physiology, Justus Liebig University Giessen

^b^ Department of Cardiac and Vascular Surgery, University Hospital Giessen and Marburg, Justus Liebig University Giessen, Germany

^c^ Department of Cardiology University Hospital Giessen and Marburg, Justus Liebig University Giessen, Germany

^d^ Department of Radiology, University Hospital Giessen and Marburg, Justus Liebig University Giessen, Germany

**Corresponding author:**

Bernd Niemann

Department of Cardiac and Vascular Surgery

University Hospital Giessen and Marburg

Justus Liebig University Giessen

Rudolph-Buchheim-Strasse 7

35392 Giessen

Germany

Phone: 0049-0641-985-56233

Fax: 0049-0641-985-44309

Email: bernd.niemann@chiru.med.uni-giessen.de

**Results**

**Characteristics of patients**

Since access route was suggested to have an impact on in-hospital outcomes, early and late mortality, we first analyzed our data regarding access strategies. Transfemoral (TF) access was used in 83.4% of the cases and transapical (TA) access in 16.6%. As shown in Table 1, TF and TA cohorts exhibited largely comparable risk profiles. TF patients tended to have a significantly higher BMI and TA patients suffered more often from three-vessel coronary artery disease (CAD) and left main stem CAD (Tab. 1). However, the grade of CAD did not differ between BMI groups. About 43% of patients in both groups suffered from reduced cardiac functional status according to the NYHA classification (NYHA III or IV) and the average EuroSCORE II was >4 % (Tab. 1). As peripheral artery disease (PAD) was a selection criterion for TA access, these patients exhibited significantly more PAD as well as carotid artery disease (Tab. 1). No significant difference between TA and TF patients was observed for risk factors/comorbidities, effective orifice area, aortic valve gradient or preprocedural clinical laboratory parameters with the exception of GFR (Tab. 1).

Perioperative morbidity

Patients with BMI > 35 kg/m² had a slightly increased morbidity, mildly prolonged **intensive care unit stay**, hospitalization or ventilatory support (p=NS; Suppl. Fig. 1B). These patients (BMI > 35 kg/m²) showed a higher inflammatory load at baseline (Suppl. Fig. 2). Maximum leucocyte count or plasma C-reactive protein (CRP) concentrations did not differ between the BMI groups perioperatively (Suppl. Fig. 2). Patients with a BMI > 35 kg/m² showed higher initial plasma lactate compared to all patients with a BMI < 30 kg/m² (Suppl. Fig. 2) and reached highest plasma lactate levels compared to all other groups during the perioperative course (Suppl. Fig. 2). Similarly, initial and perioperative GFR was lowest in severely obese patients compared to all other groups (Suppl. Fig. 2).

**Supplementary Figure 1: Operative procedures and perioperative course.**

Patients were assigned to the following subgroups: BMI < 25 kg/m², n=152, 25 kg/m²< BMI <30 kg/m², n=158 and obesity grade I (30 kg/m²< BMI <35 kg/m², n=60) and obesity grade II and III (BMI > 35 kg/m², n=33). **A:** Operative Procedure: Differences in transfemoral access, pre-dilatation prior to TAVI, need of conversion to open surgery and surgical revision between the 4 groups. **B:** Perioperative morbidity: Differences in procoagulatory medication, the occurrence of vascular complications or stroke and the necessity for implantation of a permanent pacemaker between the 4 groups. **C:** Long-term morbidity: Differences in hospitalization, stay in the intensive care unit (ICU) and duration of ventilation between the 4 groups. Data are expressed as mean ± SD or as median and 25%/75% confidence intervals if not normally distributed. Data were analyzed by one-way ANOVA followed by Tukey’s test.

**Supplementary Figure 2: Blood parameters before TAVR and during the post-interventional course.** Leucocytes, CRP, lactate and GFR prior to TAVR (upper panel) and maximum measured values during the post-interventional course (lower panel). Groups as described in Suppl. Fig. 1. Data are expressed as median and 25%/75% confidence intervals and were analyzed by one-way ANOVA followed by Tukey’s test.

**Supplementary Figure 3: Correlation between BMI or psoas muscle area and selected parameters.**

**A:** Correlation of body mass index vs. epicardial fat area, abdominal visceral fat area or abdominal subcutaneous fat area, all normalized to body surface area (BSA). **B:** Correlation of body mass index vs. psoas muscle area, BSA or age. **C:** Correlation of psoas muscle area vs. epicardial fat area, abdominal visceral fat area or abdominal subcutaneous fat area. Data from 403 TAVR patients each.

**Supplementary Figure 4: Impact of fat area on mortality and morbidity**

Impact of epicardial or abdominal visceral fat area normalized to body surface area (BSA) on survival, vascular complications or stroke. Data are expressed as mean ± SD or as median and 25%/75% confidence intervals if not normally distributed. Data were analyzed t-tests. Number of patients included 403, single measurement for each variable per patient.

**Supplementary Figure 5: Computed tomography-derived cardiac parameters**

Total heart area including epicardial adipose tissue, interventricular septum end-diastolic diameter **(IVSDd) and** total heart area normalized to body surface area (BSA). Groups as described in Suppl. Fig. 1. Data are expressed as mean ± SD or as median and 25%/75% confidence intervals if not normally distributed. Data were analyzed by one-way ANOVA followed by Tukey’s test.

**Supplementary Figure 6:**

Preoperative serum leptin in all patients (n=403) according to the BMI groups as described in Suppl. Fig. 1. Data are expressed as mean ± SD and were analyzed by one-way ANOVA followed by Tukey’s test.

**Supplementary Figure 7:**

**Age of patients at day of operation grouped according to BMI cohorts.** Data are expressed as mean ± SD and were analyzed by one-way ANOVA followed by Dunn's Method test. Groups differ significantly (p=0.001)
