# Supplementary figures and images for: Epicardial adipose tissue and muscle distribution affect outcomes in very old patients after transcatheter aortic valve replacement
Source: Eur Heart J Open. 2024 Sep 20;4(5):oeae073. doi: 10.1093/ehjopen/oeae073 (PMC11414403; doi:10.1093/ehjopen/oeae073)

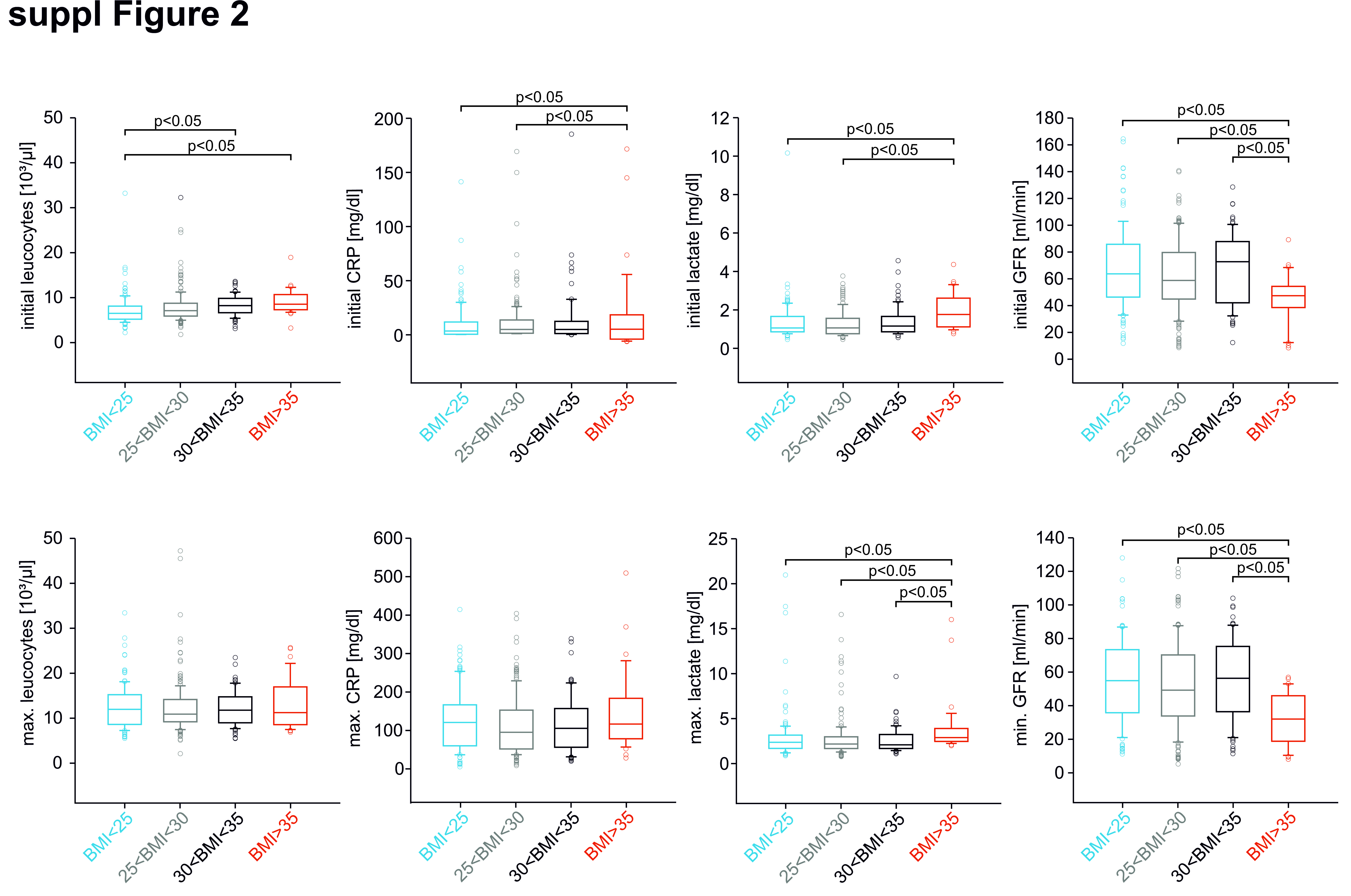

Supplement: oeae073_Supplementary_Data [file oeae073_supplementary_data.zip › suppl Figure 2.jpg]

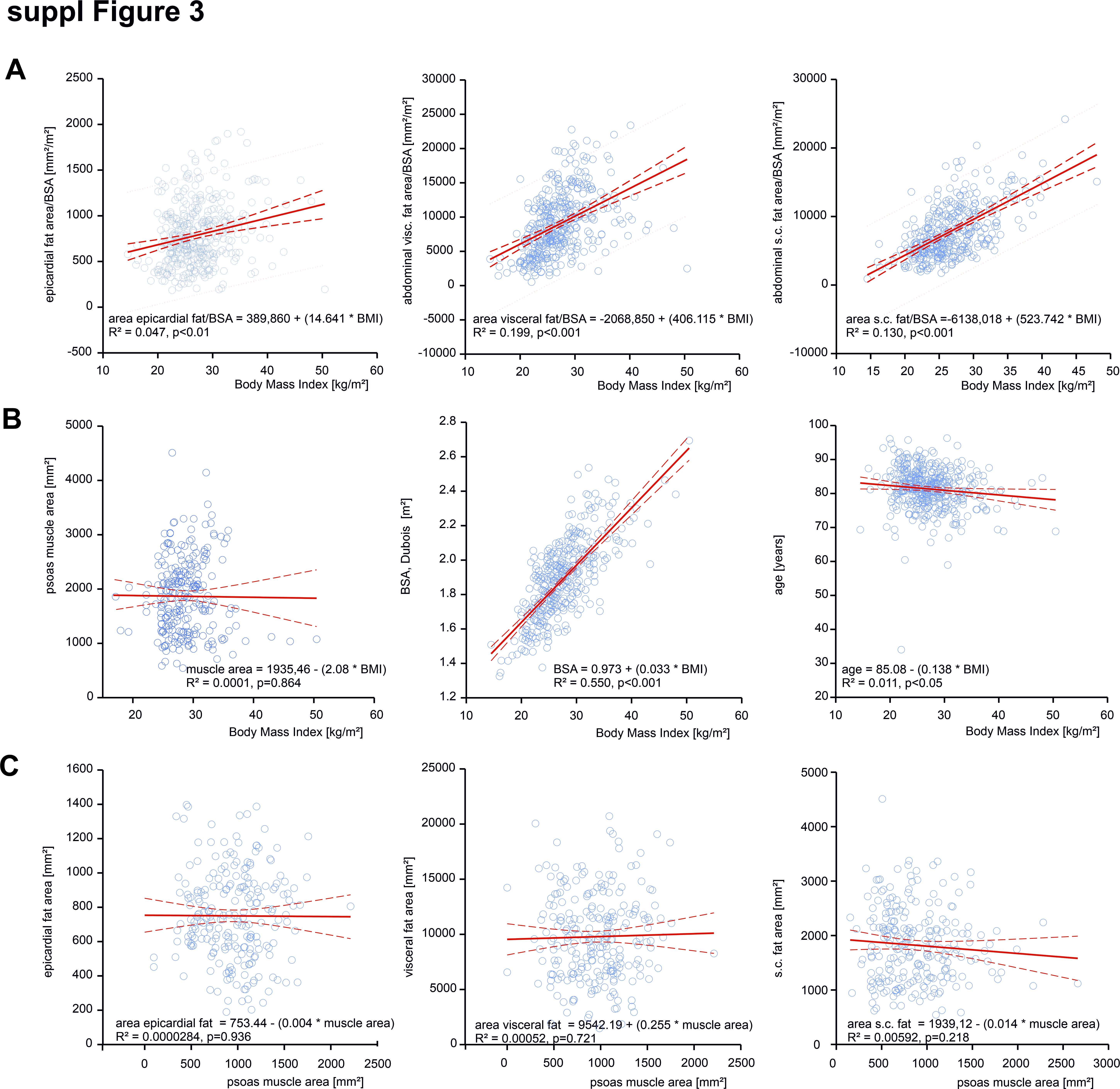

Supplement: oeae073_Supplementary_Data [file oeae073_supplementary_data.zip › suppl Figure 3.jpg]

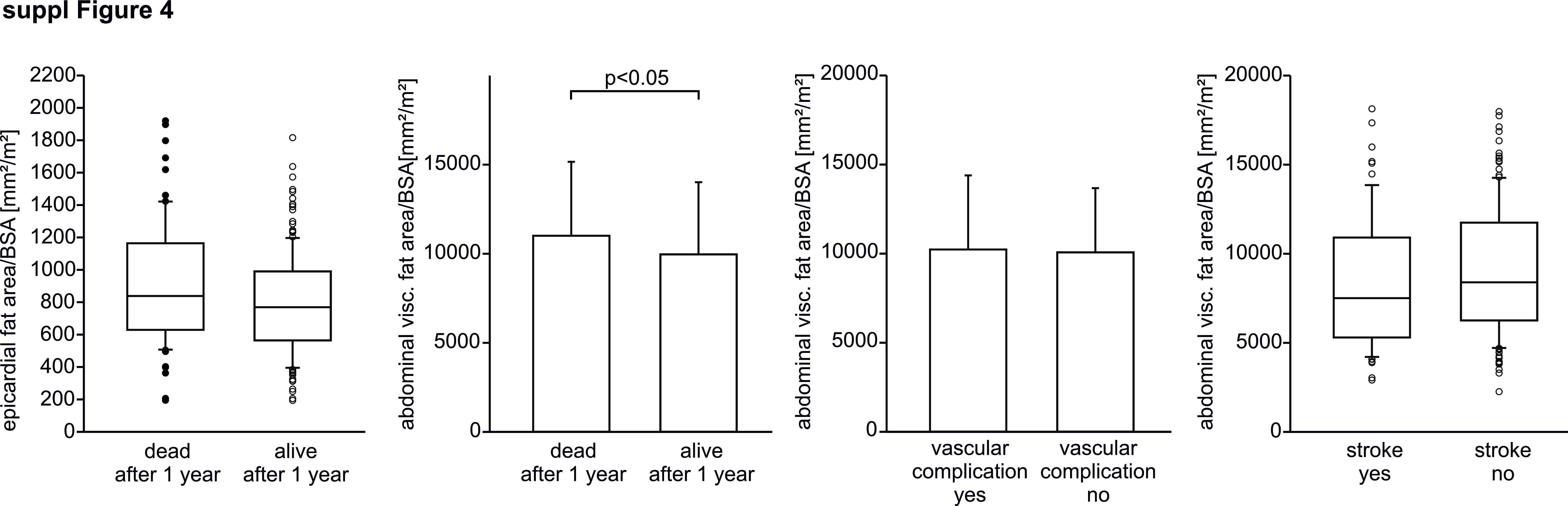

Supplement: oeae073_Supplementary_Data [file oeae073_supplementary_data.zip › suppl Figure 4.jpg]

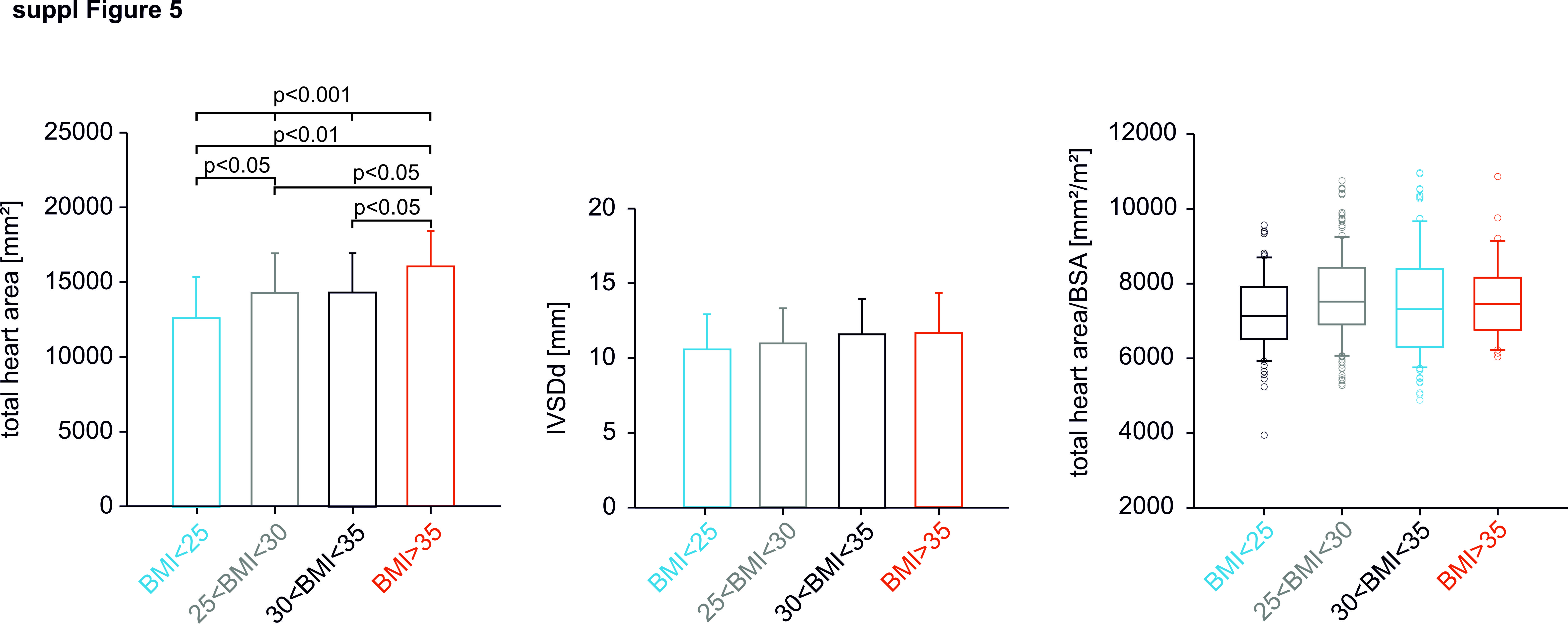

Supplement: oeae073_Supplementary_Data [file oeae073_supplementary_data.zip › suppl Figure 5.jpg]

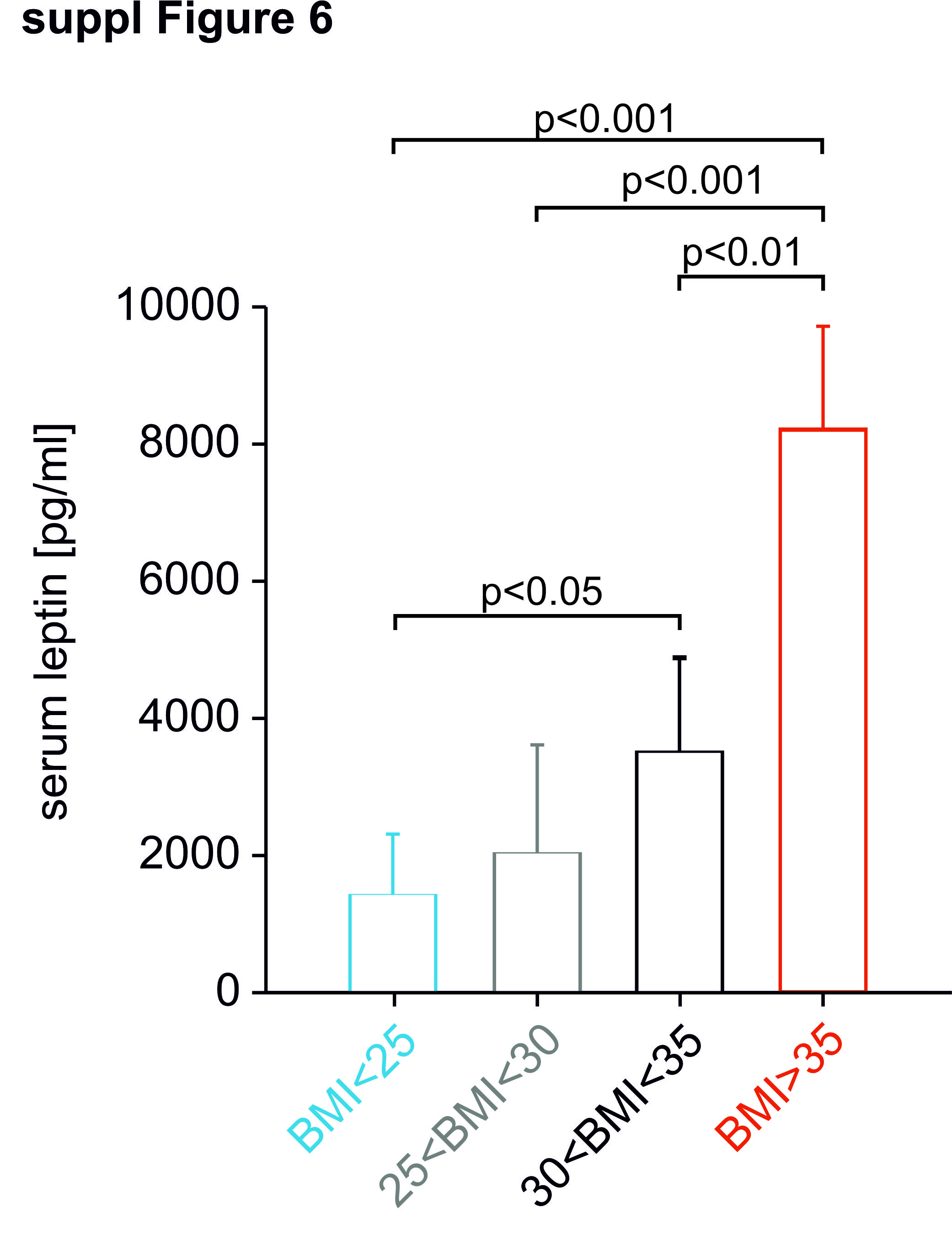

Supplement: oeae073_Supplementary_Data [file oeae073_supplementary_data.zip › suppl Figure 6.jpg]

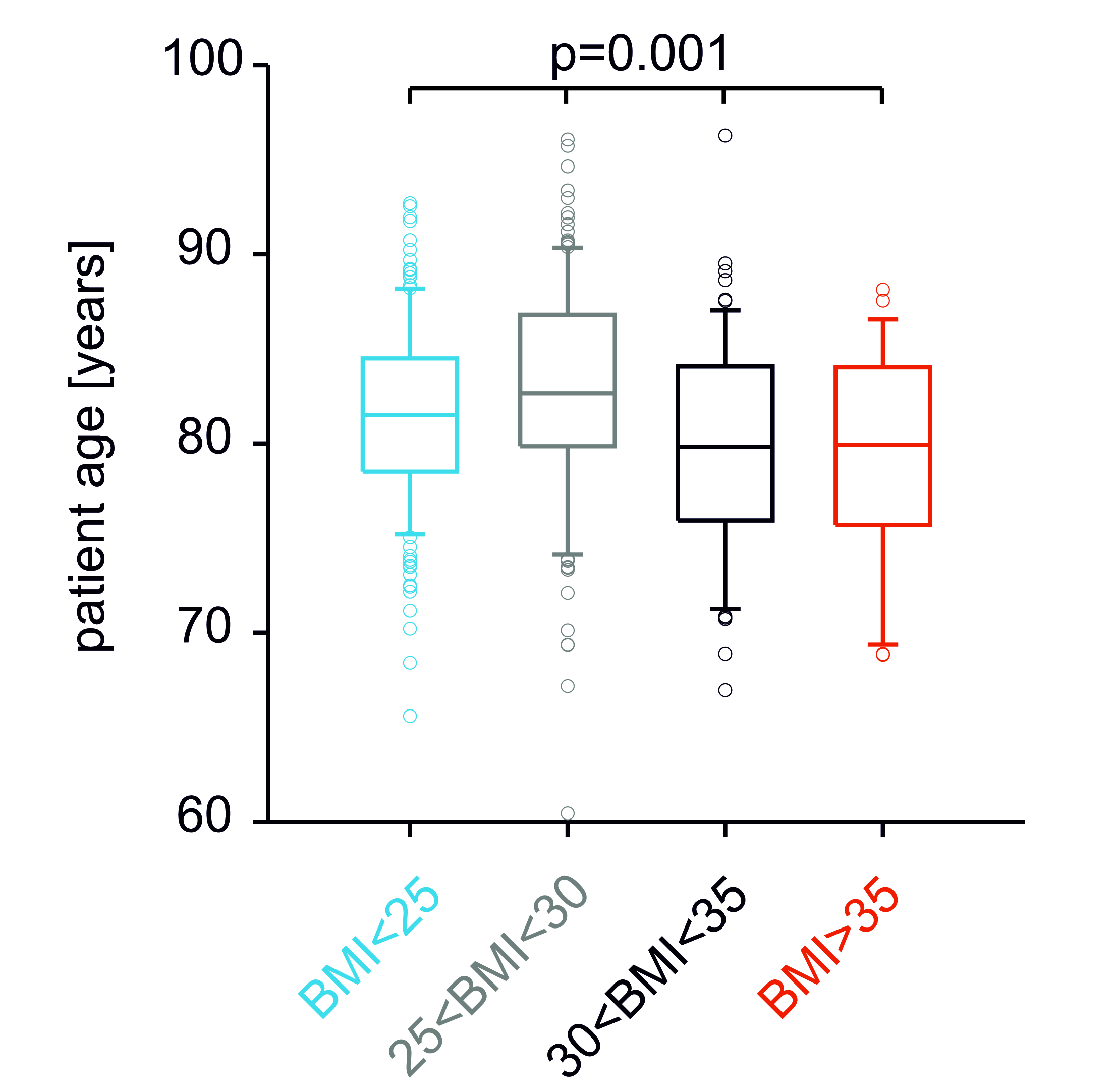

Supplement: oeae073_Supplementary_Data [file oeae073_supplementary_data.zip › suppl Figure 7 0724.jpg]
